# Cryoglobulinaemia in Egyptian Patients with Extrahepatic Cutaneous Manifestations of Chronic Hepatitis C Virus Infection

**DOI:** 10.1155/2015/182609

**Published:** 2015-12-29

**Authors:** Doaa Salah Hegab, Mohammed Abd El Rahman Sweilam

**Affiliations:** ^1^Faculty of Medicine, Dermatology and Venereology Department, Tanta University Hospitals, El Geish Street, Tanta, Gharbia Governorate 31111, Egypt; ^2^Faculty of Medicine, Clinical Pathology Department, Tanta University Hospitals, El Geish Street, Tanta, Gharbia Governorate 31111, Egypt

## Abstract

*Background*. Hepatitis C is a global major health problem with extremely variable extrahepatic manifestations. Mixed cryoglobulinaemia (MC) shows a striking association with hepatitis C virus (HCV) infection, and it is sometimes asymptomatic. The skin is a frequently involved target organ in MC.* Objective*. To investigate the prevalence of cryoglobulinaemia in a sample of Egyptian patients with cutaneous manifestations of chronic HCV infection and to correlate its presence with clinical criteria and liver function tests.* Methods*. One hundred and eighteen patients with skin manifestations of chronic compensated hepatitis C were included. Venous blood was tested for liver function tests and serum cryoglobulins.* Results*. Twelve patients (10.169%) were positive for serum cryoglobulins (2 with pruritus, 4 with vasculitic lesions, 3 with livedo reticularis, one with oral lichen, one with chronic urticaria, and another with Schamberg's disease). Vasculitic lesions and livedo reticularis of the legs showed higher prevalence in cryoglobulin-positive than in cryoglobulin-negative patients. Presence of serum cryoglobulins did not relate to patients' demographic or laboratory findings.* Conclusions*. Fortunately, MC is not markedly prevalent among Egyptians with cutaneous lesions of chronic hepatitis C, and cryopositivity was commonly, but not exclusively, detected with cutaneous vasculitis and livedo reticularis. Laboratory testing for cryoglobulins in every HCV patient is advisable for earlier MC detection and management.

## 1. Introduction

Chronic hepatitis C virus (HCV) infection is a major public health problem that affects approximately 300 million people worldwide. Egypt has the highest and devastating prevalence of HCV in the world, amounting to 14–20% [1, 2].

Patients with chronic HCV infection frequently present with extrahepatic manifestations involving different organ systems leading to the concept of systemic HCV infection. According to different studies, 40–75% of patients infected with HCV might develop at least one extrahepatic manifestation during the course of the disease, and sometimes these could represent the first and sole signal of HCV infection [3]. These manifestations include autoimmune phenomena, low-grade chronic systemic inflammation, and frank autoimmune and/or rheumatic diseases [4, 5].

There are many cutaneous manifestations of chronic HCV infection including necrolytic acral erythema, livedo reticularis, cutaneous leukocytoclastic vasculitis, porphyria cutanea tarda, pruritus, urticaria, lichen planus, polyarteritis nodosa, erythema nodosum, erythema multiforme, pyoderma gangrenosum, and mixed cryoglobulinaemia (MC) [6].

MC is a systemic vasculitis that is considered the most common extrahepatic manifestation of HCV infection. Clinical manifestations associated with MC include joint involvement, fatigue, myalgia, renal immune-complex disease, cutaneous vasculitis, and peripheral neuropathy [7, 8].

It has been postulated that MC with chronic HCV infection is commonly associated with skin involvement in the form of cutaneous vasculitis ranging from palpable purpura (leukocytoclastic vasculitis) and petechiae in the lower extremities to large necrotic non-healing ulcerations [9]; however, its association with different dermatologic manifestations in Egyptian patients with compensated chronic HCV infection was not clearly studied.

In the present study we sought to investigate the prevalence of cryoglobulinaemia in a sample of Egyptian patients with extrahepatic cutaneous affection in the setting of chronic HCV infection and to correlate its presence with clinical criteria and biochemical measures of liver function.

## 2. Materials and Methods

### 2.1. Study Participants

The study involved a group of one hundred and eighteen patients with cutaneous manifestations of chronic compensated HCV infection on follow-up treatment who were referred to the outpatient clinics of Dermatology & Venereology Department or who were collected from inpatient wards and outpatient clinics of Tropical Medicine Department in Tanta University Hospitals. HCV infection was diagnosed by anti-HCV antibodies through third generation enzyme-linked immunosorbent assay (ELISA) and HCV ribonucleic acid by polymerase chain reaction (PCR). Only patients with cutaneous affection who were positive for HCV antibodies within 6 months prior to the study were enrolled. Baseline evaluation included disease history in addition to physical general and dermatological examination. Investigative evaluation included routine biochemical panel, complete hemogram, chest X-ray, pelvic-abdominal U/S, and urine and stool analysis, in addition to liver function tests. Patients with acute hepatitis C, lymphoma, hepatocellular carcinoma, and decompensated liver cirrhosis (ascites, oesophageal varices, hepatic encephalopathy, or lower limb edema) were excluded. All included patients signed an informed written consent and the study was approved by the institutional ethical committee of Tanta University.

### 2.2. Blood Sample Collection

Fifteen mL of venous blood was drawn in Vacutainer tubes prewarmed at 37°C and left for 2 hours to clot at the same temperature; then the serum was separated by centrifugation (2500 ×g for 10 min) at 37°C. Samples were dispensed in a graduated tube for cryoglobulins assay.

### 2.3. Cryoglobulins Detection by the Traditional Method

Serum cryoglobulins were detected through the traditional method [10, 11]. The presence of precipitate in the samples was determined by visual inspection, before centrifugation and after 1, 3, 7, and 15 days of cold incubation at 4°C. Subjective evaluation of cryoprecipitate was done, and absence of cryoprecipitate was scored as negative while cryoprecipitate was scored as positive. On the 15th day of cold incubation, samples were centrifuged at 2500 ×g for 10 min at 4°C and the amount of cryoglobulins was estimated. Redissolution of the cryoglobulin precipitate by rewarming to 37°C was done. If the precipitate is not resoluble within a few minutes then the result is negative and no further analysis was adopted, while resolubilization at 37°C confirmed a positive result.

### 2.4. Statistical Analysis

Statistical presentation and analysis of the present study was conducted using the mean value, standard deviation, Student's *t*-test, or *χ* ^2^-square test (for comparison of qualitative values) by Statistics Package for Social Sciences (SPSS) version 18. Manifestations associated with cryoglobulinaemia were compared by odds ratio (OR) and 95% confidence interval (CI). For all tests a *P* value < 0.05 was considered statistically significant.

## 3. Results

Baseline demographic, clinical, and laboratory characteristics of the studied patients are shown in Table 1. Pruritus was the most common skin manifestation among the studied HCV patients. It was noted in 74 patients (62.7%) out of the 118 patients included, and the most commonly affected sites were the legs and the trunk. Vasculitic lesions were noted in 9 patients (7.627%) and appeared as palpable purpura in 5 patients, vesicles and pustules on lower limbs in 1 patient, and erythematous nodules ± ulceration on the legs in 3 patients.

Cryoglobulins were detected in the sera of 12 patients with a total of 10.169%. Among the cryopositive patients, 2 patients had persistent pruritus, 4 had leukocytoclastic vasculitic lesions on the lower limbs (3 with palpable purpura, and 1 with nodules ± ulcerations of lower limbs), 3 had livedo reticularis, one had oral lichen (erosive type), one had chronic urticaria, and another one had pigmented purpuric dermatosis presented as Schamberg's disease (Table 1).

The relationships between the presence of serum cryoglobulins and the clinical and laboratory characteristics of the patients are shown in Table 2. Cryoglobulin-positivity was not significantly related to either patients' age, sex, or any of the performed liver function tests (AST, ALT, ALP, serum albumin, total bilirubin, or prothrombin time) (*P* value > 0.05 for all).

There was a statistically significant lower association of pruritus with cryoglobulin-positivity than cryoglobulin-negativity (*P* value = 0.001), while, on the contrary, vasculitic lesions of the lower limbs and livedo reticularis were significantly more prevalent in cryoglobulin-positive than cryoglobulin-negative patients (*P* value < 0.001 for both) (Table 2).

## 4. Discussion

MC is a systemic small- and medium-sized vessels vasculitis secondary to vascular deposition of circulating immune-complexes, mainly cryoglobulins and complement [12]. The association between MC and chronic HCV infection has been well established, and unfortunately severe and life threatening complications were reported in up to 10% of MC patients in whom the mortality ranged from 20 to 80% [13]. A previous meta-analysis showed that 44% patients with chronic HCV infection had circulating immune complexes with cryoprecipitating properties [14], and it has been previously reported that the skin is the most frequently involved target organ in MC [15]. However, the association of MC with different extrahepatic manifestations and with cutaneous manifestations in particular has not yet been clearly investigated in Egyptian patients who have an extraordinarily high prevalence of HCV infection.

In our study, pruritus, leukocytoclastic vasculitic lesions (palpable purpura, vesicles, nodules ± ulceration, and crustation) of the lower extremities, and lichen planus were found to be the most frequent skin manifestations of chronic HCV infection among study participants, and that was consistent with the findings of previous studies [16–18].

In the present study, cryoglobulins were isolated in 12 patients (10.169%) out of the included 118 patients with extrahepatic cutaneous affection of HCV infection. It is noticeable that although more than half of the detected cryopositive patients had dermatological manifestations of HCV which were suggestive for cryoglobulinaemia (33.3% of cryopositive patients had vasculitic lesions on lower limbs and 25% had livedo reticularis), several patients were positive for serum cryoglobulins even in the absence of a clear clinical picture suggestive of autoimmune small vascular illness (16.6% of cryopositive patients were associated with intractable pruritus, 8.3% with oral erosive lichen planus, 8.3% with chronic urticaria, and 8.3% with Schamberg's disease). Accordingly, earlier identification of asymptomatic MC is possible through earlier detection of extrahepatic manifestations of HCV infection and particularly the dermatologic manifestations and screening those patients for the presence of serum cryoglobulins.

In a similar context, fatigue was reported to be the most frequent nonspecific clinical symptom of chronic HCV infection [19], and at the same time it is considered a part of the clinical picture of HCV-associated cryoglobulinaemia, since this symptom could be detected more frequently in cryoglobulin-positive than in cryoglobulin-negative patients with hepatitis C as described in a previous study [16]. So, even the nonspecific mild extrahepatic symptoms could act as a signal of a deeper dangerous ongoing vasculitic pathology.

In previous reports, palpable purpura was observed in 10–21% of patients with clinically manifested cryoglobulinemic syndrome [6, 20] and in 7% of all HCV infected patients [21]. In the current study, vasculitic lesions predominantly over the lower extremities were observed in 9 of the 118 included patients (7.627%), with much higher prevalence in cryoglobulin-positive than in cryoglobulin-negative patients (33.33%* versus* 4.71%, OR 10.100, 95% CI 2.255–45.219). Livedo reticularis was observed in 3 out of the 118 included patients (2.542%), and all patients with livedo were positive for serum cryoglobulins with higher prevalence in cryoglobulin-positive than in cryoglobulin-negative patients (25%* versus* 0%, *P* value < 0.001).

To the best of our knowledge, no previous reports investigating the prevalence of MC in HCV infected patients with skin manifestations were performed, but the frequency of MC in HCV-positive patients varied in the literature. Gad et al. [22] reported that the prevalence of MC in Egyptian patients infected with HCV-genotype 4 was 14%, and that was significantly lower than its prevalence in Japanese patients infected with genotype 1b (40%). It has been reported that 20% of the patients with HCV have MC, but most have cryoglobulins levels of less than 6%, which is not clinically significant and that only 3% of HCV patients have clinically significant MC with cryoglobulins levels of more than 6% [23, 24]. The mean cryocrit levels in HCV patients were determined to be about 2% [25].

Our study detected a lower prevalence of MC among Egyptian patients with cutaneous manifestations of HCV infection (10.169%) than expected. These findings suggest that cryoglobulinaemia in the context of hepatitis C may be less prevalent in Egypt than in other areas and this could be attributed to (1) the apparent protective role of* Schistosoma mansoni* coinfection, which is common in Egypt, against the development of immune-mediated diseases such as MC in chronic HCV-infected patients [26]. Helminthic infections are characterized by a strong T helper-2 response as well as an overall downregulated immune system which are both beneficial in protecting the host from developing autoimmune diseases and/or relieving symptoms of an established autoimmune disease [27]. (2) There are HCV-related factors, particularly genotype where HCV genotype 4 is the most prevalent in Egypt (90%), while genotype 1 is the most prevalent worldwide followed by genotype 3 (affecting 46.2% and 30.1% of global HCV cases, resp.) [28, 29]. (3) MC shows higher incidence and significantly higher levels of cryoglobulins (>6%) in patients with hepatic cirrhosis and later stages of fibrosis [30], while all the participants in our study were having chronic compensated HCV infection. (4) Different cryoglobulins detection methods from one study to another and difficult detection of cryoglobulins because of their thermolability might cause a heterogeneity in the prevalence results between studies. (5) Finally, there are environmental and/or host genetic factors that might contribute to the pathogenesis of MC [31].

Actually, it is still unknown why only some chronically infected HCV patients develop MC and only some of these exhibit systemic symptoms (MC syndrome). Several studies have investigated the pathogenetic basis of MC and suggested that the virus is able to trigger such a disorder only in the presence of genetic factors that are still unknown [32]. The data that were reported are heterogeneous and sometimes even conflicting. There is a complex relationship between HCV-related MC and the host's genetic background including HLA polymorphisms (as HLA-A9, HLA-B8, HLA-DR3, HLA-DR11, and HLA-DR5-DQ3), cytokine mutations (affecting IL-10 promoter (-1082GG), or BAFF promoter (-871T)), genetic variability of IgG Fc receptors, fibronectin polymorphisms (called* Msp*I and* Hae*IIIb), and low serum levels of vitamin D associated with* CYP27B1* AC or CC genotype [32–37].

The results of the current study detected no significant relation between the presence of serum cryoglobulins in study participants on one side and patients' age, sex, or biochemical parameters of liver functions on the other side.

Some authors had reported that cryoglobulinaemic vasculitis was significantly more common in female population with HCV infection [38], although this association was nonsignificant in another report [39]. Abbas et al. [26] reported that cryoglobulinaemia in their Egyptian patients with HCV was not related to age or progression of cirrhosis; meanwhile it was negatively correlated with serum ALT and serum AST levels, and it was positively correlated with female gender. The discrepancy in the results could be due to variation in sample size and inclusion criteria among different studies.

## 5. Conclusions

In conclusion, although Egypt still has the highest prevalence of HCV infection worldwide, fortunately, MC is not markedly prevalent among Egyptian patients with cutaneous manifestations of chronic HCV and that might indicate that Egypt has a lower incidence of MC than other areas. Although a large proportion of cryopositive cases with cutaneous manifestations of HCV present with vasculitic lower limb lesions or livedo reticularis, still some patients with MC could be either asymptomatic or present incidentally with nonspecific lesions. Laboratory testing for cryoglobulins is usually neglected in clinical practice and we think that it should be adopted as a routine test for HCV infected patients even in absence of suggestive manifestations of vasculitis, and that might help in earlier detection and management of MC with HCV before the occurrence of serious internal insult.

## Figures and Tables

**Table 1:** Clinical and laboratory criteria of HCV infected patients with cutaneous manifestations (*n* = 118).

Variable	Value in study participants
Age range in years, mean (SD)	24–63, 47.1 (7.5)
Sex, M (%)/F (%)	84 (71.2%)/34 (28.8%)
Liver function test range, mean (SD)	
AST (IU/L)	12–168, 62.6 (32.4)
ALT (IU/L)	10–177, 61 (30.5)
ALP (IU/L)	51–266, 109.6 (34.6)
Serum albumin (g/dL)	3.5–4.8, 4.1 (0.4)
Total serum bilirubin (mg/dL)	0.5–6.4, 1 (0.9)
Prothrombin time	11–18, 13.2 (1)
Skin manifestations of chronic HCV *n* (%), +ve/−ve cryoglobulins	
Pruritus	74 (62.7%), 2/72
Vasculitic lesions	9 (7.6%), 4/5
Lichen planus (oral ± cutaneous)	8 (6.8%), 1/7
Lichen planus (cutaneous only)	3 (2.5%), 0/3
Melasma	6 (5.1%), 0/6
Urticaria	4 (3.4%), 1/3
Livedo reticularis	3 (2.5%), 3/0
Necrolytic acral erythema	3 (2.5%), 0/3
Erythema nodosum	2 (1.7%), 0/2
Prurigo nodularis	2 (1.7%), 0/2
Vitiligo	2 (1.7%), 0/2
Pigmented purpuric dermatosis (Schamberg's disease)	1 (0.8%), 1/0
Psoriasis	1 (0.8%), 0/1

ALP: alkaline phosphatase; ALT: alanine aminotransferase; AST: aspartate aminotransferase.

**Table 2:** Relationship between cryoglobulin-positivity and patients' characteristics.

Characteristic	Cryoglobulin-positive patients (*n* = 12)	Cryoglobulin-negative patients (*n* = 106)	*P* value (Student's *t* test or *χ* ^2^)	
Age range in years, mean (SD)	29–63, 49.1 (7.1)	24–59, 47.4 (5.1)	0.24	
Gender, *n* (%)				
Male (*n* = 84)	9 (75%)	75 (70.8%)	0.76	
Female (*n* = 34)	3 (25%)	31 (29.3%)		
Liver function test range, mean (SD)				
AST (IU/L)	28–77, 44.9 (10.6)	12–168, 65.5 (14.6)	0.1	
ALT (IU/L)	31–73, 42.9 (16.5)	10–177, 63.9 (31.3)	0.1	
ALP (IU/L)	92–266, 131 (37.1)	51–200, 96.3 (5.2)	0.1	
Serum albumin (g/dL)	3.6–3.9, 4 (0.5)	3.5–4.8, 4.1 (0.6)	0.7	
Total serum bilirubin (mg/dL)	0.47–1.36, 1.1 (0.7)	0.5–6.4, 1 (0.9)	0.1	
Prothrombin time	11–15.6, 13.2 (0.6)	11–18, 13.3 (1)	0.6	

Cutaneous extrahepatic manifestations associated with MC, *n* (%)			*P* value (*χ* ^2^)	Odds ratio (95% confidence)

Pruritus (*n* = 74)	2 (16.6%)	72 (67.9%)	0.001^*∗*^	0.09 (0.02–0.5)
Vasculitic lesions of lower limbs (*n* = 9)	4 (33.3%)	5 (4.7%)	<0.001^*∗*^	10.1 (2.3–45.2)
Oral lichen planus (*n* = 8)	1 (8.3%)	7 (6.6%)	0.82	1.29 (0.1–11.4)
Chronic urticaria (*n* = 4)	1 (8.3%)	3 (2.8%)	0.32	3.12 (0.3–32.6)
Livedo reticularis (*n* = 3)	3 (25%)	0	<0.001^*∗*^	—
Pigmented purpuric dermatosis (*n* = 1)	1 (8.3%)	0	0.003^*∗*^	—

MC: mixed cryoglobulinemia; ^*∗*^significant.
